# Validation of prognostic scores for survival in cancer patients beyond first-line therapy

**DOI:** 10.1186/1471-2407-11-95

**Published:** 2011-03-15

**Authors:** Olivier Trédan, Isabelle Ray-Coquard, Gisèle Chvetzoff, Paul Rebattu, Agathe Bajard, Sylvie Chabaud, David Pérol, Chadi Saba, Florent Quiblier, Jean-Yves Blay, Thomas Bachelot

**Affiliations:** 1 Université de Lyon, Centre Léon Bérard, Department of Medical Oncology, 28 rue Laennec, 69008 Lyon, France; 2 Université de Lyon, Centre Léon Bérard, Department of Statistics, UBET, 28 rue Laennec, 69008 Lyon, France; 3 Université de Lyon, Centre Léon Bérard, Department of Clinical Studies, 28 rue Laennec, 69008 Lyon, France

## Abstract

**Background:**

We aimed to validate prognostic scores for survival in patients undergoing chemotherapy for advanced or metastatic cancer after first-line treatment.

**Methods:**

We previously described two models with good prognostic value based on a combination of Performance Status (PS) and either lactate dehydrogenase (LDH) level or lymphocyte count. These factors were evaluated for their ability to predict overall survival (OS) in a prospective cohort of 299 patients. Clinical and blood parameters were prospectively recorded. Candidate prognostic factors for OS with 0.05 significance level in univariate analysis were included in a multivariate Cox model.

**Results:**

Median age was 59 years (range: 26-85). Primary tumor sites were breast (45%), lung (15%), ovaries (11%) and others (29%). The number of metastatic sites was 1 (29%), 2 (48%), >2 (23%). Median follow-up and median OS were 12 and 6 months, respectively. Multiple regression analysis confirmed that PS >1, lymphocyte count ≤700/μL and LDH >600 UI/L were independent predictors of short OS, as well as interleukin 6 (IL-6) level, serum albumin concentration and platelet count.

**Conclusions:**

Prognostic scores using PS plus LDH level or PS plus lymphocyte count were validated for predicting survival in metastatic cancer patients in relapse beyond first-line treatment. A score combining PS, LDH, lymphocyte and platelet count, serum albumin and IL-6 level was superior in determining patients' prognosis.

## Background

Prediction of survival for patients with advanced cancer who have already received first-line treatment is critical to decision making regarding subsequent treatments. Models allowing accurate estimation of life expectancy are needed to make a more informed treatment decision, in particular to guide decisions about chemotherapy in vulnerable patients. Doctors' survival predictions for palliative patients are often optimistic [1,2]. The Research Network of the European Association for Palliative Care (EAPC) has provided evidence-based clinical recommendations for prognosis in patients with advanced cancer [3]. One of the six key recommendations is that physicians should systematically use prognostic scores to stratify patients into groups with different survival times. One of the prognostic tools specifically considered by the working group was the Palliative Prognostic (PaP)score based on Performance Status (PS), the presence or absence of dyspnea and anorexia, white blood cell counts, and the clinician's prediction of survival. It has been validated successfully in patients admitted to the oncology ward of a university teaching hospital [4]. However, the PaP score may be limited in that it was developed in the 1990 s for patients with far-advanced cancer referred to community hospitals.

We aimed to develop a novel prognostic scoring system for patients undergoing chemotherapy. Predictive models for survival integrating combinations of clinical and biological factors have been reported [5-10]. Biological characteristics such as lactate dehydrogenase (LDH) level [6], lymphocyte count [5,7], interleukin 6 (IL-6) level [8,9], or vascular endothelial growth factor (VEGF) level [10] have been correlated with poor outcome. The primary endpoint of this study was the validation of two prognostic scores that we have previously shown to be correlated with survival in a Cox proportional hazards regression model. These scores were based on PS plus LDH level (score A) or PS plus lymphocyte count (score B). Both scores have been shown to stratify patients into groups with significantly different survival prognosis [6,7]. We also aimed to investigate a third model including additional clinical and biological characteristics and thus potentially allowing for more accurate prognostication.

## Methods

This was a prospective, observational, single-centre study.

### Patients

Inclusion criteria were: patients >18 years old with locally advanced or metastatic cancer who had received at least one line of systemic treatment (chemotherapy or immunotherapy) for metastatic disease. Enrolment was proposed after failure of the line of treatment being administered, when decision was made to shift to a different treatment. Patients were excluded from the study if they were not covered by the French health insurance system. Written informed consent was obtained from each patient. The institutional ethics committee (named CCPPRB Lyon-B) approved the study protocol before implementation, on March 17, 2000.

### Data

The case report form of the study was designed to collect the following parameters: age, gender, Eastern Cooperative Oncology Group (ECOG) PS, weight loss (> 10% of the initial body weight in the 6 months before study inclusion), primary diagnosis, number and sites of metastases, previous and current anticancer treatments, interval between diagnosis, first recurrence and study inclusion, and quality of life (QoL) assessed by the patients themselves on a 0-10 visual analog scale (VAS). Laboratory tests included complete blood counts (hemoglobin, neutrophils, lymphocytes, platelets), albumin, LDH and C-reactive protein (CRP) levels. Serum samples were collected and stored at -80°C. Commercially available immunoassay kits were used according to the manufacturers' instructions in order to determine IL-6 and Vascular Endothelial Growth Factor (VEGF) levels [8,9]. According to our previous studies [6-10], the cutoff of each parameter was albumin: 38 g/L, lymphocytes: 700 μL^-1^, LDH: 600 U/L, IL-6: 8 pg/mL, and VEGF: 755 pg/mL. As for CRP, several studies in different cancer sites have demonstrated its prognostic value with a cutoff at 10 mg/L [11-13].

### Statistical considerations

#### Sample size

In retrospective cohorts from our institution, 20% of metastatic cancer patients died within three months after the beginning of the chemotherapy. With 300 patients enrolled, we expected to have a 73% power to detect a hazard ratio of 1.5 in the final model [14].

#### Survival analysis

the primary outcome was overall survival, defined as the time from inclusion to date of death or date of last follow-up for patients alive at last contact. Survival distributions in prognostic groups were estimated by the Kaplan-Meier method.

#### Univariate and multivariate analyses

to evaluate the relationship between survival and baseline characteristics, all clinical and biological variables were included in univariate Cox proportional hazard regression models. For validation of scores A and B, parameters from each score were entered in multivariate Cox models. Furthermore, candidate prognostic factors for OS with a 0.05 level of significance in univariate analysis were entered in a multivariate Cox model. A backward selection procedure was then used to build a third model. All relevant interactions were included in the model and then non-significant ones were removed step by step to obtain the final model. Based on the final Cox proportional hazard model, a prognostic score was computed for each patient. It consisted in the sum of the baseline variables multiplied by the estimated coefficients for these baseline variables. Participants were grouped in quantiles of the prognostic score corresponding to quantiles of risk. Overall survival curves were then plotted depending on the quantile of risk.

#### Quantification of the performance of each score

to test whether the addition of parameters significantly improves the goodness of fit, comparisons between scores were performed using a likelihood ratio test (LRT). This test applies to nested models and is based on the LRT statistic calculated from the likelihood scores of the two models to be compared. The LRT statistic follows a chi-square distribution, with degrees of freedom equal to the number of additional parameters in the most complex model. Lower likelihood scores indicate a better fitted model. In addition, similarly to the report by Chow *et al *[15], the C index proposed by Harrell *et al *[16] and the D-statistic of Royston and Sauerbrei [17] were calculated for the 3 models as measures of discrimination. The C index is the probability that, for a randomly chosen pair of patients, the predicted and observed outcomes are concordant (i.e. the patient having the best outcome is the one having the best predicted outcome). A value of 0.5 indicates no predictive discrimination and a value of 1.0 indicates perfect separation of patients with different outcomes. We also calculated a C index corrected for possible overfitting using the bootstrap method, which is the bias-corrected C index [16]. This index is a better estimate of how well the model will discriminate prognosis in the future. The D-statistic was also used as a measure of discrimination of the survival models because of its ability to stratify the risk of death among groups of patients. The larger the D statistic, the greater the degree of separation in a prognostic model.

All statistical analyses were realized using SAS^® ^v.9.1 (Cary, NC, USA).

## Results

### Patient characteristics

Between 2000 and 2005, 300 patients treated in our institution (Léon Bérard Comprehensive Cancer Centre, Lyon, France) for locally advanced or metastatic cancer and who had failed first-line treatment were enrolled in the study. In our institution, most patients receive more than two lines of chemotherapy, which is covered by the French public health insurance system. One patient was excluded from the analysis because he was enrolled before first-line chemotherapy.

The characteristics of the 299 patients analyzed are summarized in Table 1. Two hundred and twenty patients (74%) were female. Median age was 58.6 years (range 26-85). Many patients (132 of 299; 45%) presented with metastatic breast cancer; 132 (44%) had PS > 1. Only 47 patients (16%) suffered from locally advanced cancer without metastasis. Sixty-three (22%) patients had presented with a weight loss of 10% or more in the previous 6 months. On a 0 to 10 scale (VAS), the median QoL score as self-assessed by the patients was 5 (range: 0-10); 25% of the patients presented with VAS <3.5. The median time intervals between initial diagnosis and inclusion, and between first recurrence and inclusion were 39 months (range: 0.8-472 months) and 17 months (range: 0-148 months), respectively.

**Table 1:** Patient characteristics

Patient Characteristics	Number (Percentage)
Total	299 (100)

Age (years)	
Median [range]	59 [26-85]

Gender	
Male	79 (26)
Female	220 (74)

ECOG Performance Status	
0	33 (11)
1	134 (45)
> 1	132 (44)

Weight loss >10% (in the past 6 months)	63 (22)
Missing data	9

Primary tumor	
Breast	132 (45)
Lung	45 (15)
Ovarian	34 (11)
Head and neck	18 (6)
Colorectal	12 (4)
Soft tissue	11 (4)
Other	47 (16)

Number of metastatic sites	
1	86 (29)
2	139 (46)
> 2	74 (25)

Site of metastases	
Liver	143 (50)
Lung	132 (46)
Bone	88 (31)
Soft tissue	69 (24)
Brain	25 (9)
Skin	9 (3)

Previous treatment	
Adjuvant chemotherapy	119 (42)
Immunotherapy	29 (10)
Treatment completed	131 (49)
First-line	70 (26)
Second-line	64 (24)
> Second-line	34
Missing data	

Treatment after inclusion	
Chemotherapy	293 (98)
Immunotherapy	5 (2)
Radiation therapy	3 (1)†
Supportive care	4 (1)

† patients may have received combination therapies

Biological variables are shown in Table 2. Complete blood count was available for 295 patients (99%). Other laboratory data were missing due to incomplete collection of blood samples by the referring physicians. A majority of patients presented with anemia (58%), low serum albumin (70%), elevated CRP (> 10 mg/L; 59%). Ninety-six (32%) patients had lymphopenia ≤700/μL and 32 (11%) had thrombopenia ≤130 G/L. The median time interval between the last chemotherapy and study inclusion was 1.1 month, both for the entire cohort and for patients with lymphopenia ≤700/μL. Respectively 130 (48%) and 66 (24%) patients had IL-6 >8 pg/mL and VEGF >755 pg/mL.

**Table 2:** Blood tests

Parameters	Number (Percentage)
Hemoglobin	
Abnormal (< 11.5 g/dL for women; <13.0 g/dL for men)	170 (58)
Missing data	4

Absolute neutrophil count	
< 2000/μL	16 (5)
2000-7500/μL	225 (76)
> 7500/μL	54 (18)
Missing data	4

Lymphocyte count	
≤700/μL	96 (32)
700-1000/μL	63 (21)
≥1000/μL	137 (46)
Missing data	3

Platelet count	
< 130 G/L	32 (11)
130-400 G/L	231 (78)
> 400 G/L	32 (11)
Missing data	4

Albumin	
< 38 g/L	172 (70)
Missing data	52

LDH	
> 600 U/L	89 (36)
Missing data	48

CRP	
≤10 mg/L	92 (41)
10-50 mg/L	79 (35)
> 50 mg/L	54 (24)
Missing data	74

IL-6	
> 8 pg/mL	133 (48)
Missing data	21

VEGF	
> 755 pg/mL	66 (24)
Missing data	20

LDH: lactate dehydrogenase; CRP: C-reactive protein; IL-6: interleukin 6; VEGF: Vascular Endothelial Growth Factor

### Survival

The median follow-up was 11.9 months. Two hundred and sixty-four patients (89%) were dead at the date of evaluation. Median OS was 6.4 months (95% CI, 5.6-7.2).

### Validation of the two pre-existing prognostic scores

**Figure 1:**
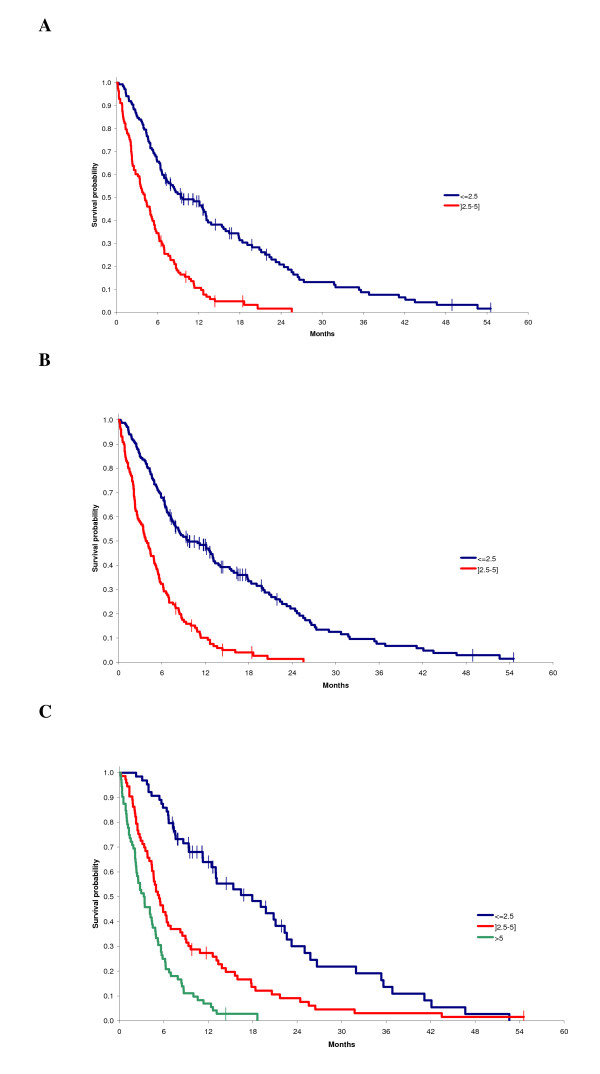
**Analysis of quantiles of the prognostic score corresponding to quantiles of risk with Kaplan-Meier overall survival analysis and Cox proportional hazard regression model, using score A with ECOG performance status and lactate dehydrogenase level (Figure 1A), score B with ECOG performance status and lymphocyte count (Figure 1B), or ECOG performance status, lactate dehydrogenase level, lymphocyte count, serum albumin and platelet count (Figure 1C)**. Participants with risk >5 were pooled. No participant was in this group with score A or B.

The prognostic value for overall survival of score A, with PS >1 (HR = 2.52 [95% CI, 1.87-3.40]) and LDH >600 U/L (HR = 1.80 [95% CI, 1.34-2.42]), and score B, with PS >1 (HR = 2.80 [95% CI, 2.13-3.68]) and lymphocytes ≤700/μL (HR = 1.69 [95% CI, 1.30-2.20]), was validated in the study cohort (Figure 1A and 1B).

**Figure 2:**
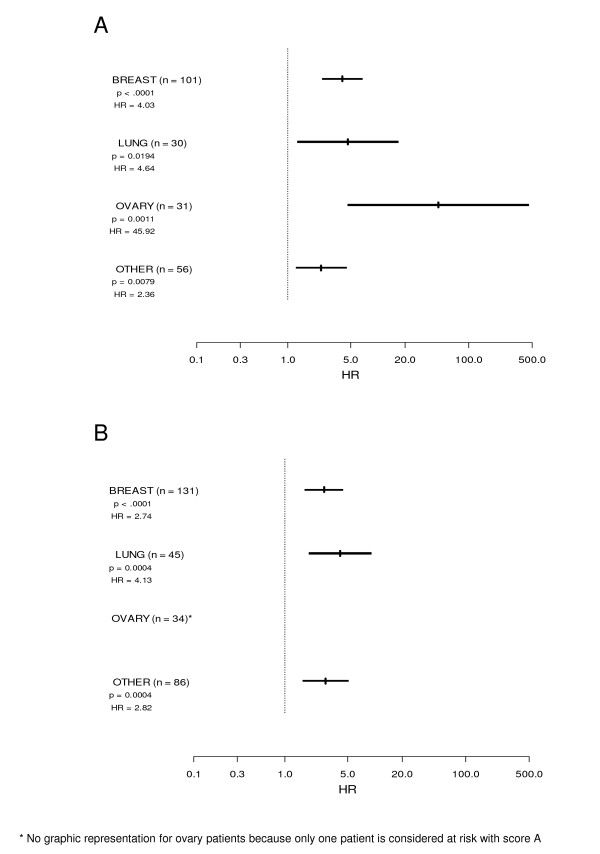
**Results of Cox proportional hazard regression model in the most common types of primary tumors, using score A with ECOG performance status and lactate dehydrogenase level (Figure 2A), and score B with ECOG performance status and lymphocyte count (Figure 2B)**.

Scores A and B were validated for the most common types of primary tumors (Figure 2A and 2B).

### Building of a more accurate prognostic score

In univariate analysis, 12 of 16 variables were found to be significant at a 5% level (Table 3). In multivariate analysis (209 patients), 6 variables were associated with a significantly poorer prognosis: PS >1, IL-6 >8 pg/mL, LDH >600 U/L, lymphocytes ≤700/μL, albumin < 38 g/L and platelets < 130 G/L (Table 4). Only PS >1 was associated with a more than two-fold increased hazard ratio of death. Interactions between type of cancer and each of the 5 significant variables were tested in the multivariate model using a backward selection procedure: no interaction was found to be significant in the final model. Interestingly, a forward selection has been also applied to the data, resulting in the same final model.

**Table 3:** Prognostic parameters on univariate analysis

	Univariate analysis		
	HR	95% CI	p value
ECOG Performance Status			
0-1			
> 1	2.95	[2.27-3.84]	< .0001

Platelet count (G/L)			
≥130			
< 130	2.67	[1.83-3.88]	< .0001

CRP (mg/L)			
≤10			
> 10	2.25	[1.67-3.04]	< .0001

LDH (U/L)			
≤600			
> 600	2.17	[1.64-2.88]	< .0001

IL-6 (pg/mL)			
≤8			
> 8	2.16	[1.66-2.81]	< .0001

Albumin (g/L)			
≥38			
< 38	2.12	[1.56-2.89]	< .0001

Hemoglobin			
Normal			
Abnormal†	1.88	[1.46-2.43]	< .0001

Weight loss >10% (in the past 6 months)			
No			
Yes	1.83	[1.37-2.45]	< .0001

Lymphocyte count			
> 700/μL			
≤700/μL	1.80	[1.39-2.33]	< .0001

VEGF (pg/mL)			
≤755			
> 755	1.55	[1.15-2.09]	0.0044

Number of metastatic sites			
≤2			
> 2	1.50	[1.14-1.99]	0.0042

Quality of life using 0-10 VAS			
≥5			
< 5	1.53	[1.18-1.98]	0.0012

Absolute neutrophil count			
≥2			
< 2	1.30	[0.78-2.16]	0.3

Liver metastases			
No			
Yes	1.19	[0.93-1.53]	0.16

Age (years)			
≤60			
> 60	0.97	[0.76-1.23]	0.8

Previous adjuvant chemotherapy			
No			
Yes	0.91	[0.71-1.18]	0.5

LDH: lactate dehydrogenase; CRP: C-reactive protein; IL-6: interleukin 6; VEGF: Vascular Endothelial Growth Factor; VAS: visual analog scale.

† < 11.5 g/dL for women, <13.0 g/dL for men

**Table 4:** Prognostic parameters on multivariate analysis (209 patients)

	Multivariate analysis		
	HR	95% CI	p value
ECOG Performance Status			
0-1			
> 1	2.07	[1.48-2.91]	< 0.0001

IL-6 (pg/mL)			
≤8			
> 8	1.72	[1.25-2.36]	0.0009

LDH (U/L)			
≤600			
> 600	1.60	[1.16-2.21]	0.0045

Lymphocyte Count			
> 700/μL			
≤700/μL	1.43	[1.04-1.95]	0.0268

Albumin (g/L)			
≥38			
< 38	1.47	[1.02-2.11]	0.0374

Platelet count (G/L)			
≥130			
< 130	1.70	[1.02-2.81]	0.0402

LDH: lactate dehydrogenase; IL-6: interleukin 6

According to the quantiles of risk based on the final cox proportional hazrard model, low risk (≤ 2.5; 31%), medium risk (between 2.5 and 5; 35%) and high risk (> 5; 34%) were associated with median OS of 18, 5 and 4 months, respectively. The different survival curves are shown in Figure 1C.

Focusing on breast cancer patients (Table 5), 3 prognostic factors remained independently predictive of overall survival: PS >1 (HR = 2.08 [95% CI, 1.30-3.35]), LDH >600 U/L (HR = 2.47 [95% CI, 1.56-3.92]) and IL-6 >8 pg/mL (HR = 1.83 [95% CI, 1.17-2.86]).

**Table 5:** Prognostic parameters found significant on multivariate analysis for 103 breast cancer patients

	Multivariate analysis		
	HR	95% CI	p value
LDH (U/L)			
≤600			
> 600	2.47	[1.56-3.92]	0.0001

ECOG Performance Status			
0-1			
> 1	2.08	[1.30-3.35]	0.0025

IL-6 (pg/mL)			
≤8			
> 8	1.83	[1.17-2.86]	0.0079

LDH: lactate dehydrogenase

### Quantification of score performance

Results of the LRT when scores A and B were tested in the current cohort were 2002 and 2425, respectively. The D statistics were 0.93 and 0.97 and the C indexes 0.66 [95% CI, 0.63-0.69] and 0.67 [95% CI, 0.64-0.70] for scores A and B, respectively.

Using the new prognostic score combining the 6 significant variables (PS, LDH, lymphocytes, IL-6, albumin and platelets), the LRT was 1577, which is better than with either score A or B (p < 0.0001). Similarly, the D statistic was 1.23, which is in favour of a better predictive discrimination than with either score A or B. The C index was 0.72 [95% CI, 0.68-0.76], which indicated good concordance between predicted and observed values. Furthermore, the bias-corrected C index obtained from 1000 bootstrap samples was 0.71.

## Discussion

In this prospective study, both score A (PS plus LDH level) and score B (PS plus lymphocyte count) were validated as suitable for identifying distinct risk groups with different durations of survival. Furthermore, we showed that the combination of PS and biological covariates such as LDH, lymphocyte and platelet counts, serum albumin and IL-6 levels is an effective strategy to predict survival for patients with advanced or metastatic cancer receiving further treatment after the first-line. Our third model including all six parameters was successfully validated according to the different methods used. Notably, the bias-corrected C index that was used as an alternative for external validation confirmed the good performance of the model. However, this validation study has several limitations. The percentage of colorectal cancer patients in our population study is low (3.7%), which may represent a selection bias. Also, there are several missing biochemical parameters, especially CRP (25%). Furthermore, regarding the CRP cutoff, we have tested it at 10 mg/L, which may appear to be low for advanced cancer patients, and may explain why CRP was not statistically significant in our multivariate analysis. However, several studies have already reported data on cancer patients with this cutoff [11-13].

The decision to stop chemotherapy is one of the hardest challenges in oncology practice. Chemotherapy remains widely prescribed for terminally ill patients despite side effects and poor efficacy [18,19]. We aimed to develop prognostic scores that would help clinicians estimate patients' survival regardless of initial tumor site. Therefore, we validated our prognostic scores in different cancer patient populations (Figure 2A and 2B). We acknowledge that, although cancer treatment is known to be cancer-type dependent, we did not include in our analysis prognostic factors specific to each cancer type. However, our scores are of importance and of potential clinical relevance since they may help the physicians discuss palliative treatment options with the patients and their families. Prognostication is not simply prediction of response to therapy, and some prognostic factors, such as PS or lymphocyte count, are useful across different tumor types, as we and others have already shown [4,10,15,20,21]. Furthermore, clinical or pathological data such as tumor grade or node involvement can reasonably be used as prognostic factors in early-stage disease. After first-line treatment failure, these data do not seem suitable for predicting short-term prognosis [22,23].

Several clinical signs have been shown to be prognostically important in terminally ill cancer patients. For example, dyspnea, constipation, dizziness, pain, anxiety or depression are individually related to life expectancy, but their prognostic value has rarely been confirmed in multivariate analyses [20,24,25]. Therefore, our group developed 2 different PS-based prognostic scores using unbiased biological variables (scores A and B). Score B, described by Ray-Coquard *et al *[7], uses lymphopenia as a predictor of early death after chemotherapy. The threshold level of 700 lymphocytes per μl was chosen because it predicts hematological toxicity in patients who receive chemotherapy, as demonstrated in previous studies [5,7,26,27]. We recently showed that lymphopenia, with a lymphocyte count of <1000/μl, is also an independent prognostic factor for overall and progression-free survival in several cancers [21]. Score A was published by Bachelot *et al *[6]. Their study included 154 patients with solid tumors enrolled in phase I clinical trials. PS >1 and high serum LDH level (> 600 U/L) were identified as independent prognostic factors for OS. Several studies have shown that elevated LDH is significantly associated with survival in patients with terminal cancer [28-31]. Bozcuk *et al *and Suh *et al *have set the threshold for high LDH level at 378 U/L and 313 U/L, respectively [29,31]. However, reasons for patient admission in Bozcuk's study included life-threatening situations, whereas Suh's study included only patients who had been admitted to the palliative care unit.

Most studies show that PS (assessed by the Karnofsky Performance Scale or by the ECOG scale) correlates with duration of survival [3,6,7,20,24,25]. Recently, Kikuchi *et al*. have shown the benefit of adding biological markers such as serum albumin, LDH level, platelet and lymphocyte count to predict survival in terminally-ill cancer patients [32]. Serum albumin concentration is a well-known independent predictor of mortality risk [33]. In the current study, we also confirmed the prognostic value of the pro-inflammatory cytokine IL-6 [8,9,34]. However, IL-6 level is not clinically available and the usefulness of routine IL-6 assessment should be further investigated. Also, in our study we decided to use dichotomous variables that make clinical interpretation easier (the risk is either present or absent). However, we acknowledge that strong prognostic marker could be better included as linear parameter to take advantage of its prognostic information over the whole range of potential cutoffs. This might have enabled the calculation of a potentially more powerful prognostic algorithm.

## Conclusions

In conclusion, we have validated two prognostic scores (score A: PS plus LDH level, and score B: PS plus lymphocyte count) and confirmed that combination of PS and biological covariates such as serum albumin concentration, LDH level, lymphocyte count, thrombocytopenia and IL-6 level is an effective strategy to predict survival for patients with advanced or metastatic cancer receiving further treatment after first-line failure. Because prediction of survival is variable among physicians, adding more objective measures would improve the accuracy of the score and facilitate treatment decision making in metastatic cancer patients, regardless of initial tumor site.

## Competing interests

The authors declare that they have no competing interests.

## Authors' contributions

OT designed the research, collected and analyzed data, wrote the manuscript; IR treated the patients, analyzed the data and contributed to writing the manuscript; GC, PR and CS treated the patients and collected the data; AB, SC and DP performed the statistical analysis and contributed to writing the manuscript; FQ collected the data; JYB and TB designed the research, treated the patients, analyzed the data and contributed to writing the manuscript.

All authors have read and approved the final version of the manuscript.

## Pre-publication history

The pre-publication history for this paper can be accessed here:

http://www.biomedcentral.com/1471-2407/11/95/prepub
